# Supplementary material for: HR3/RORα-mediated cholesterol sensing regulates TOR signaling
Source: Nat Commun. 2026 Mar 30;17:4609. doi: 10.1038/s41467-026-71059-x (PMC13199448; doi:10.1038/s41467-026-71059-x)
Supplement: Supplementary file 1 — Supplementary Information [file 41467_2026_71059_MOESM1_ESM.pdf]

Supplementary Information

HR3/RORα-mediated cholesterol sensing regulates TOR signaling

Mette Lassen[1], Keith Pardee[2,3], Ivan Bradic[4], Lisa H. Pedersen[1], Olga Kubrak[1], Nadja Ahrentløv[1], Sebastian Clancy[2,3], Takashi Koyama[1], Aleksandar Necakov[5], Suya Liu[6], Arnis Kuksis[7,8], Gilles Lajoie[6], Aled Edwards[7,9], Aurelio A. Teleman[10], Martin R. Larsen[4], Henry M. Krause[7,8], Michael J. Texada[1], Kim Rewitz[1,*]

[1] Department of Biology, University of Copenhagen, Copenhagen, Denmark.
[2] Department of Pharmaceutical Sciences, Leslie Dan Faculty of Pharmacy, University of Toronto, Toronto, Ontario, Canada.
[3] Department of Mechanical and Industrial Engineering, University of Toronto, Toronto, Ontario, Canada.
[4] Department of Biochemistry and Molecular Biology, University of Southern Denmark, Odense, Denmark.
[5] European Molecular Biology Laboratory, Heidelberg, Germany.
[6] Department of Biochemistry, University of Western Ontario, London, Ontario, Canada.
[7] Banting and Best Department of Medical Research, Department of Molecular Genetics, University of Toronto, Toronto, Ontario, Canada.
[8] Department of Biochemistry, University of Toronto, Toronto, Ontario, Canada.
[9] The Donnelly Centre for Cellular & Biomolecular Research, University of Toronto, Toronto, Ontario, Canada.
[10] German Cancer Research Center (DKFZ), Division B140, Heidelberg, Germany.

*Correspondence: Kim.Rewitz@bio.ku.dk

Supplementary Figure 1

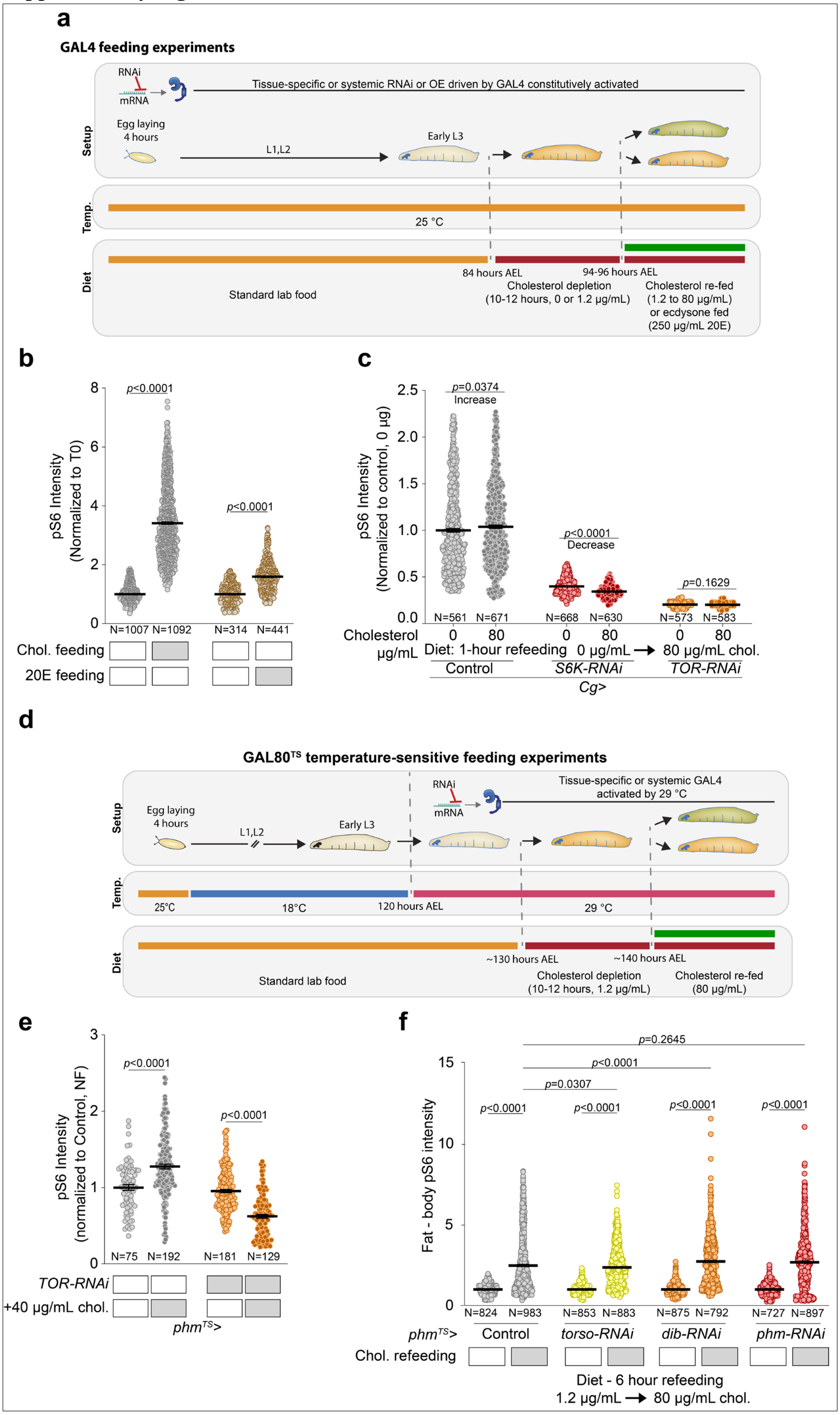

Supplementary Figure 1 (previous page). a, Schematic overview of the experimental feeding paradigm. Larvae were raised on standard lab food until 84 h after egg laying (AEL), then transferred to synthetic food containing either 0 µg/mL cholesterol or 1.2 µg/mL cholesterol for 10 h to induce cholesterol depletion. At 94 h AEL, larvae were re-fed for 0-6 h with synthetic diets supplemented with different cholesterol concentrations (1.2–80 µg/mL) or with 20-hydroxyecdysone (20E; 250 µg/mL, supplied with 1.2 µg/mL cholesterol) to examine dose-dependent activation of the TOR pathway. This scheme corresponds to the experimental setup described in the Results section. **b**, Quantification of wild-type larval fat-body pS6 response to 6 hours' feeding with cholesterol (80 µg/mL) or 20-hydroxyecdysone (20E; 250 µg/mL with 1.2-µg/mL cholesterol) following 10-hour low-cholesterol pre-feeding (1.2 µg/mL). **c**, Phospho-S6 staining of fat-body tissue from controls and animals expressing fat-body-specific (*Cg*>) knock-down of *S6K* or *TOR*, after 1 hour's refeeding with 0 or 80 µg/mL-cholesterol medium following 10-hour cholesterol deprivation (0 µg/mL cholesterol in synthetic medium). **d**, Schematic of the $GAL80^{TS}$ induction paradigm used for temporally restricted PG-specific knockdown experiments. Larvae were raised at 18 °C to keep $GAL80^{TS}$ active and thereby repress *phm-GAL4*-driven UAS-transgene expression. At 120 h AEL, larvae were shifted to 29 °C, inactivating $GAL80^{TS}$ and permitting GAL4-mediated induction of *UAS-RNAi* specifically during late larval stages. This allows TOR-pathway manipulation to be restricted to a defined developmental window prior to cholesterol refeeding (1.2 µg/mL for 10 h followed by 80 µg/mL for 6 h). **e**, Phospho-S6 response in prothoracic gland (PG) cells of control larvae and animals expressing PG-specific *TOR* knockdown, chronically fed NutriFly (NF) or NutriFly supplemented with 40 µg/mL cholesterol. **f**, Fat-body pS6 after PG-specific knockdown (phm^{TS}>) of *torso*, *phantom* (*phm*), or *disembodied* (*dib*). At 120 h AEL, knockdown was induced; larvae were placed on 1.2 µg/mL cholesterol for 10 h, then re-fed for 6 h with 1.2 or 80 µg/mL cholesterol. Statistics: (b-c,f). All data plotted as means ±SEM, normalized to control cholesterol-starvation condition (T0). Each data point represents pS6 intensity in the cytoplasm of a single cell. *P*-values calculated by Kruskal-Wallis ANOVA tests (two-sided) with Dunn's multiple comparisons. Source data are provided in the Source Data file.

Supplementary Figure 2

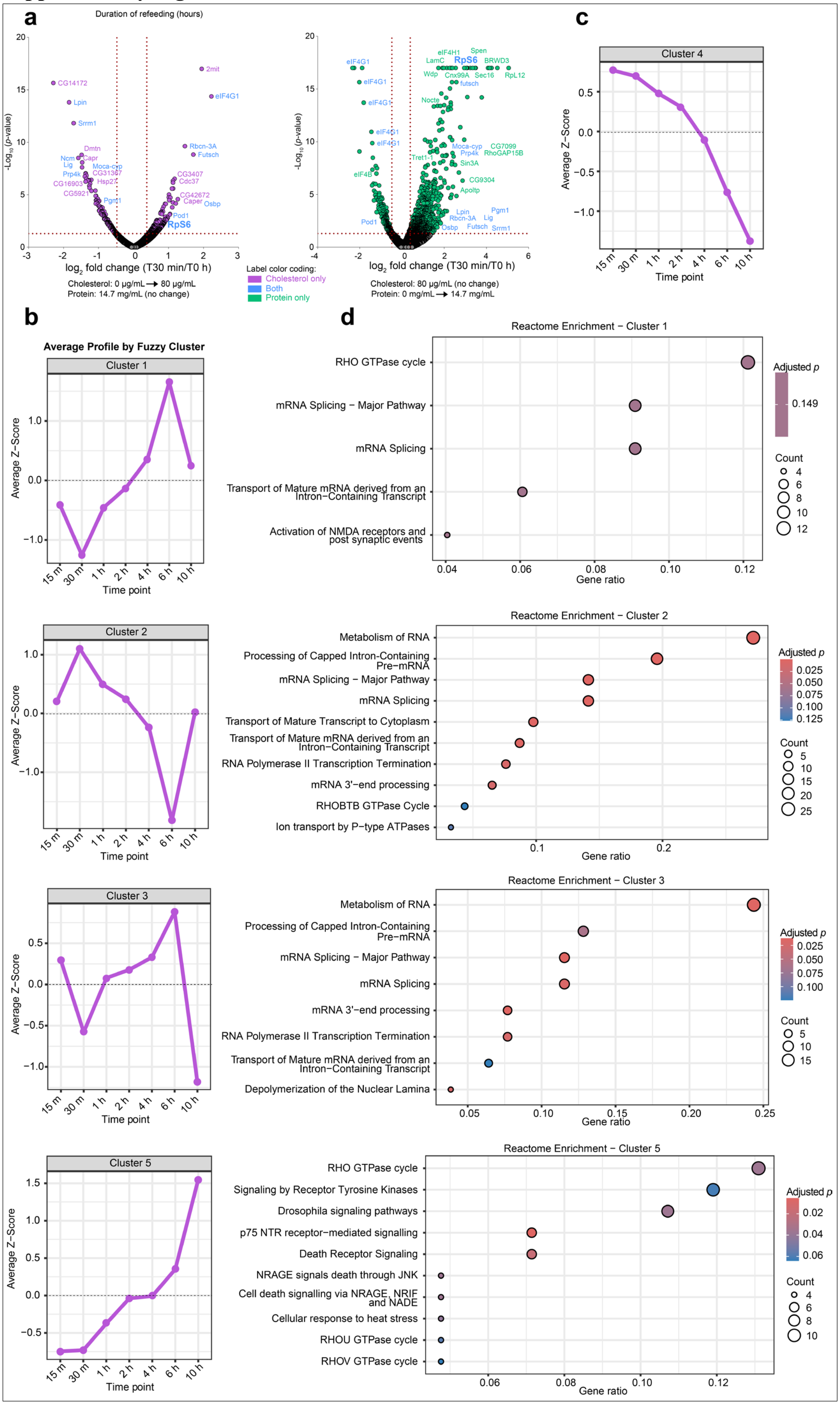

Supplementary Figure 2 (previous page). **a**, Volcano plot of phosphoproteomic changes after 30 minutes' cholesterol or protein refeeding after depletion. **b-d**, Cholesterol refeeding induces distinct clusters of similarly temporally controlled changes, presented as change over time along with pathway enrichment. **b**,**c**, Average temporal profiles of the five phosphosite clusters identified via fuzzy *c*-means clustering for cholesterol refeeding. The *y* axis displays the mean Z-score standardized abundance, and lines represent the consensus trend for all phosphosites within each cluster. **d**, Functional over-representation analysis (ORA) of the identified clusters against the Reactome database. Dot plots show the ten most significantly enriched biological pathways for each cluster. Dot size is proportional to the number of genes associated with each pathway (Count), and the color gradient indicates statistical significance (FDR-adjusted p value; FDR < 15%). Cluster 4 showed no significant Reactome enrichment under this cutoff. A phosphorylation change was considered significant with a 30% fold change ($\log_2$ FC >0.379 or <-0.515) and $p<0.05$ ($-\log_{10} p>1.3$). Source data are provided in the Source Data file.

Supplementary Figure 3

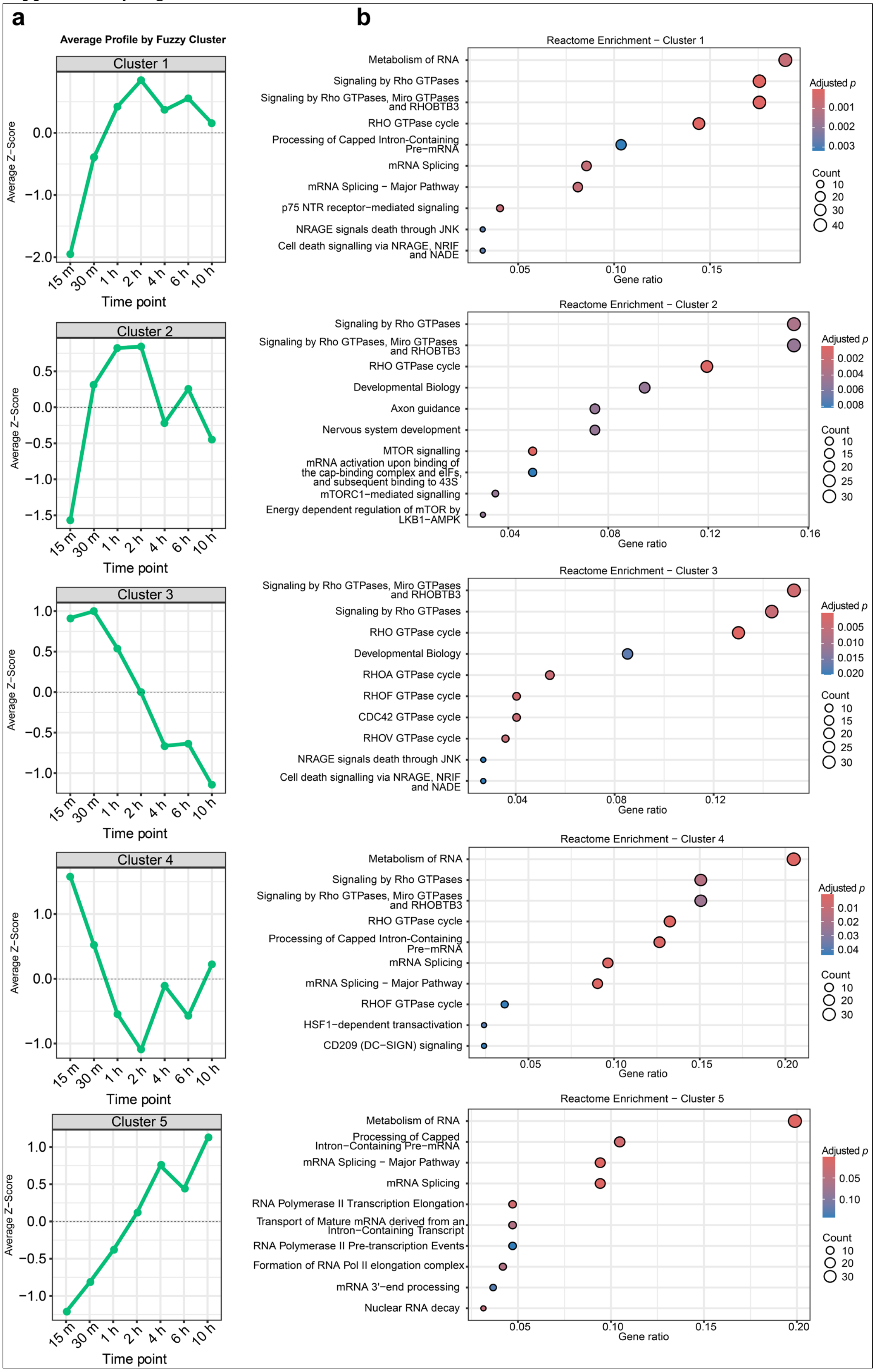

Supplementary Figure 3 (previous page). Protein refeeding induces distinct temporal phosphosite clusters and pathway enrichment. **a**, Average temporal profiles of the five phosphosite clusters identified via fuzzy *c*-means clustering for protein refeeding. The *y* axis displays the mean Z-score standardized abundance, and lines represent the consensus trend for all phosphosites within each cluster. **b**, Functional over-representation analysis (ORA) of the identified clusters against the Reactome database. Dot plots show the ten most significantly enriched biological pathways for each cluster. Dot size is proportional to the number of genes associated with each pathway (Count), and the color gradient indicates statistical significance (FDR-adjusted p value < 15%). A phosphorylation change was considered significant with a 30% fold change ($\log_2$ FC >0.379 or <-0.515) and $p<0.05$ ($-\log_{10} p>1.3$). Source data are provided in the Source Data file.

Supplementary Figure 4

a

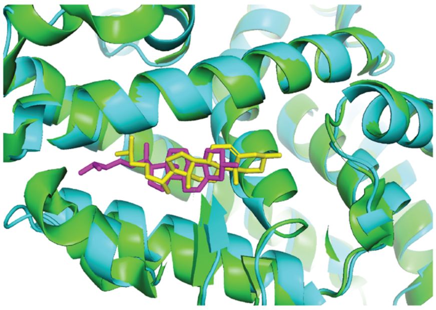

b

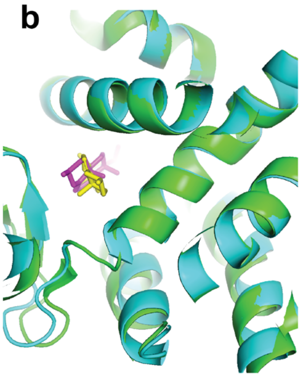

c

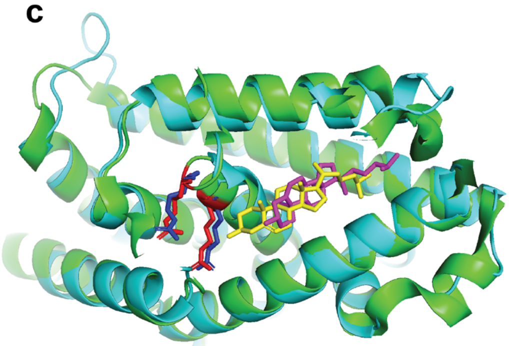

e

Peak (m/z)	Assignment (as labeled)	z	Calculated neutral mass (Da)	Δ mass vs apo (Da)
2879.38	HR3	11	31,662.10	0
2883.17	Ac-HR3	11	31,703.79	41.69
2914.56	HR3:Chol.	11	32,049.08	386.98
2919.32	Ac-HR3:Chol.	11	32,101.44	439.34

Neutral mass calculated as **M = (m/z × z) − (z × 1.0073)**, with **z = 11**.
Reference masses: **acetylation ≈ 42.01 Da**, **cholesterol ≈ 386.65 Da**.

d

```
HR3 - Isoform F - NP_001246236.1
RORα - P35398

HR3      ----------------------MYTQRMFDMWSSVTSKLEAHANNLGQSNVQSPAGQNNS   1-38
RORα     MESAPAAPDPAASEPGSSGADAAAGSRETPLNQESARKSEPPAPVRRQSYSSTSRGISVT   1-60
                                  .*   : .. : * *. *    **  .:. * . :

HR3      SGSIKAQIEIIPCKVCGDKSSGVHYGVITCEGCKGFFRRSQSSVVNYQCPRNKQCVVDRV   39-98
RORα     KKTHTSQIEIIPCKICGDKSSGIHYGVITCEGCKGFFRRSQQSNATYSCPRQKNCLIDRT   61-120
         . : .:********:*******:******************.* ..*.***:*:*::**.

HR3      NRNRCQYCRLQKCLKLGMSRDAVKFGRMSKKQREKVEDEVRFHRAQMR-----AQSDAAP   99-153
RORα     SRNRCQHCRLQKCLAVGMSRDAVKFGRMSKKQRDSLYAEVQKHRMQQQQRDHQQQPGEAE   121-180
         .*****:******* :*****************:.:  **: ** * :      *.. *

HR3      DSSVYDTQTPSSSDQLHHNNYNSYSGGYSNNEVGYGSPYGYSASVTP--------QQTMQ   154-205
RORα     PLTPTYNISANGLTELHDDLSNYIDGHTPEGSKADSAVSSFYLDIQPSPDQSGLDINGIK   181-240
           :   . :...  :**.:  *  .*  .:.. . .:  .:  .: *         : ::

HR3      YDISADYVDSTTYEPRSTIIDPEFISHADGDINDVLIKTLAEAHANTNTKL-EAVHDMFR   206-264
RORα     PEPICDYTPASGFFPYCSFTNGETSPTVSMAELEHLAQNISKSHLETCQYLREELQQITW   241-300
          :  .**. :: : * .:: : *  . ..    : * :.::::* :*   * * ::::

HR3      KQPDVSRILYYKNLGQEELWLDCAEKLTQMIQNIIEFAKLIPGFMRLSQDDQILLLKTGS   265-321
RORα     QTFLQEEIENYQNKQREVMWQLCAIKITEAIQYVVEFAKRIDGFMELCQNDQIVLLKAGS   301-360
         :    ..*  *:*  :* :*  ** *:*: ** ::**** * ***.*.*:***:***:**

HR3      FELAIVRMSRLLDLSQNAVLYGDVMLPQEAFYTSDSEEMRLVSRIFQTAKSIAELKLTET   322-381
RORα     LEVVFIRMCRAFDSQNNTVYFDGKYASPDVFKSLGCEDF--ISFVFEFGKSLCSMHLTED   361-418
         :*:.::**.* :* .:*:* :..   . :.* : ..*::  :* :*: .**:..::***

HR3      ELALYQSLVLLWPERNGVRGNTEIQRLFNLSMNAIRQELETNHAPLKGDVTVLDTLLNNI   382-441
RORα     EIALFSAFVLMSADRSWLQEKVKIEKLQQKIQLALQHVLQKNHREDG----ILTKLICKV   419-474
         *:**:.::**: .:*. :: :.:*::* :    *::: *:.**        :* .*: ::

HR3      PNFRDISILHMESLSKFKLQHPNVV---FPALYKELFSIDSQQDLT---   442-483
RORα     STLRALCGRHTEKLMAFKAIYPDIVRLHFPPLYKELFTSEFEPAMQIDG   475-523
         ..:* :.  * *.*  **  :*::*   **.******: : :  :

Sequence identity across the full alignment: 31.4%
DNA-Binding Domain (DBD) sequence identity: 77.6%
Ligand-Binding Domain (LBD) sequence identity: 28.3%
Remaining amino acids sequence identity: 12.2%
```

Supplementary Figure 4. a-c, Structural comparison of cholesterol placement in human RORα and the predicted *Drosophila* HR3 ligand-binding domain. **a**, Close-up view of the ligand-binding pocket showing cholesterol positioned within the RORα LBD (green) and the Boltz-2–predicted HR3 LBD model (cyan). Cholesterol molecules are shown as stick models (RORα, yellow; HR3 model, magenta). **b**, Alternative orientation and view of the RORα and predicted HR3 LBD structures highlighting the location of the ligand-binding pocket and cholesterol placement. **c**, Superposition of RORα (green) and the predicted HR3 LBD model (cyan) illustrating

overall structural alignment. Cholesterol is shown in yellow (RORα) and magenta (HR3 model). Arginine residues corresponding to R328 and R331 in HR3 (blue) and R367 and R370 in RORα (red) are shown as sticks to indicate their positions relative to the ligand. **d**, Sequence alignment of HR3 isoform F and human RORα (P35398). DNA-Binding Domain (DBD) is indicated in blue, while the Ligand-Binding Domain is indicated in orange. HR3 arginine residues R328 and R331 (dark blue residues in LBD) and RORα R367 and R370 (red residues in LBD) are conserved, suggesting a conserved mechanism of interaction with the 3β-hydroxyl group of cholesterol. Conserved residues between HR3 and RORα critical for cholesterol binding due to their proximity to the ligand (≤4 Å) are indicated in purple with a * below, including strictly conserved (using RORα numbering) W320, C323, K326, R367, M368, V379, F391 and H484. A lilac background indicates residues whose mutation has been shown to alter RORα function[1,2]. Residues with conserved hydrophobicity properties but with an aliphatic residue replacement in HR3 are also indicated in green, including I327L, A330M, V364L, F381Y, F399M, and V403I, and Y380L. Hydrogen bonding between conserved residues H484 and Y507 has been shown to be critical for cholesterol binding in RORα. **e**, Calculated neutral masses corresponding to the labeled m/*z* peaks in Fig. 3d,e are reported here and confirm that the mass difference between apo HR3 and the liganded species is consistent with cholesterol. Source data are provided in the Source Data file.

Supplementary Figure 5

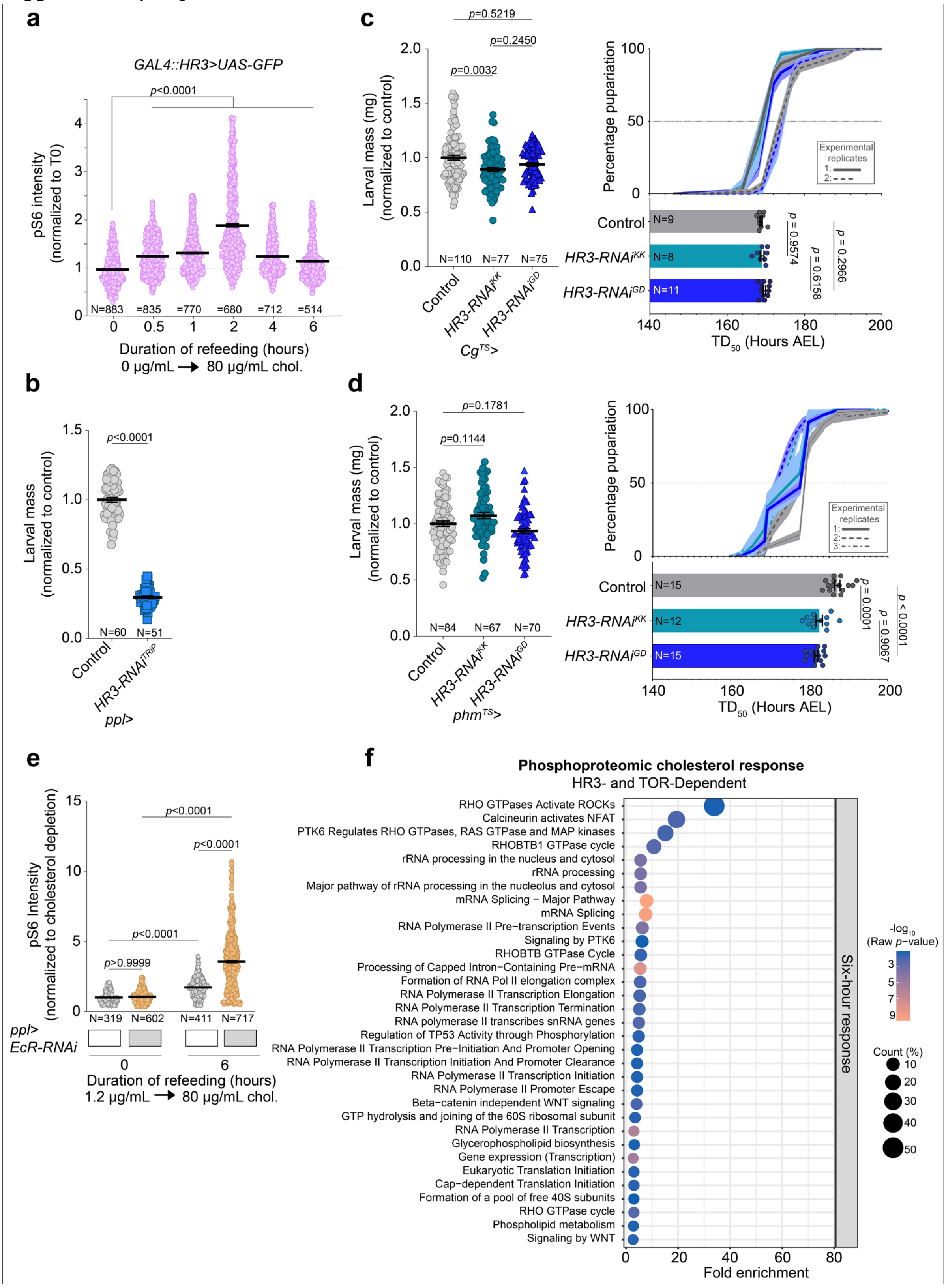

Supplementary Figure 5. a, *Drosophila* larval fat-body anti-pS6 in the same animals as in Fig. 3l in response to cholesterol re-feeding (80 µg/mL) following 10 hours' cholesterol deprivation. **b**, Larval mass at 96 hours AEL of controls and animals expressing an additional *HR3* knock-down construct in the fat body using *ppl>*, fed on STD-FF. **c**,**d** Larval mass and timing of pupariation after fat-body-specific *HR3* knockdown (c) or PG-specific *HR3* knockdown (d), induced using the temperature-sensitive Cg^{TS}> fat-body driver or phm^{TS}> PG driver by shifting larvae to 29 °C at early L3 (120 hours AEL at 18 °C). Larval weight was measured after 24 hours of RNAi

induction in the mid–third instar. e, Fat-body pS6 response to cholesterol refeeding (80 µg/mL cholesterol) in controls and larvae expressing *EcR* knock-down in the fat-body with *ppl*>, following 10-hour feeding with low-cholesterol (1.2 µg/mL) medium. Each data point reflects the cytoplasmic pS6 intensity of a single cell. **f**, Selected pathways enriched in proteins exhibiting phosphoproteomic responses to 6-hour cholesterol refeeding that were dependent on both HR3 and TOR. Pathway analysis was conducted using Panther and Reactome. Additional pathways are listed in Supplementary Data 4. Statistics: Data plotted as means ± SEM. (a) Data are normalized to T0. Each data point represents a single cell measurement. (a,c.d) Significance determined by Kruskal-Wallis nonparametric ANOVA (two-sided) with Dunn's multiple comparisons. (b) *P*-value calculated using two-tailed unpaired *t*-test. (d) Only pathways with $p<0.05$ by Fisher's *t*-test are included. Source data are provided in the Source Data file.

Supplementary Figure 6

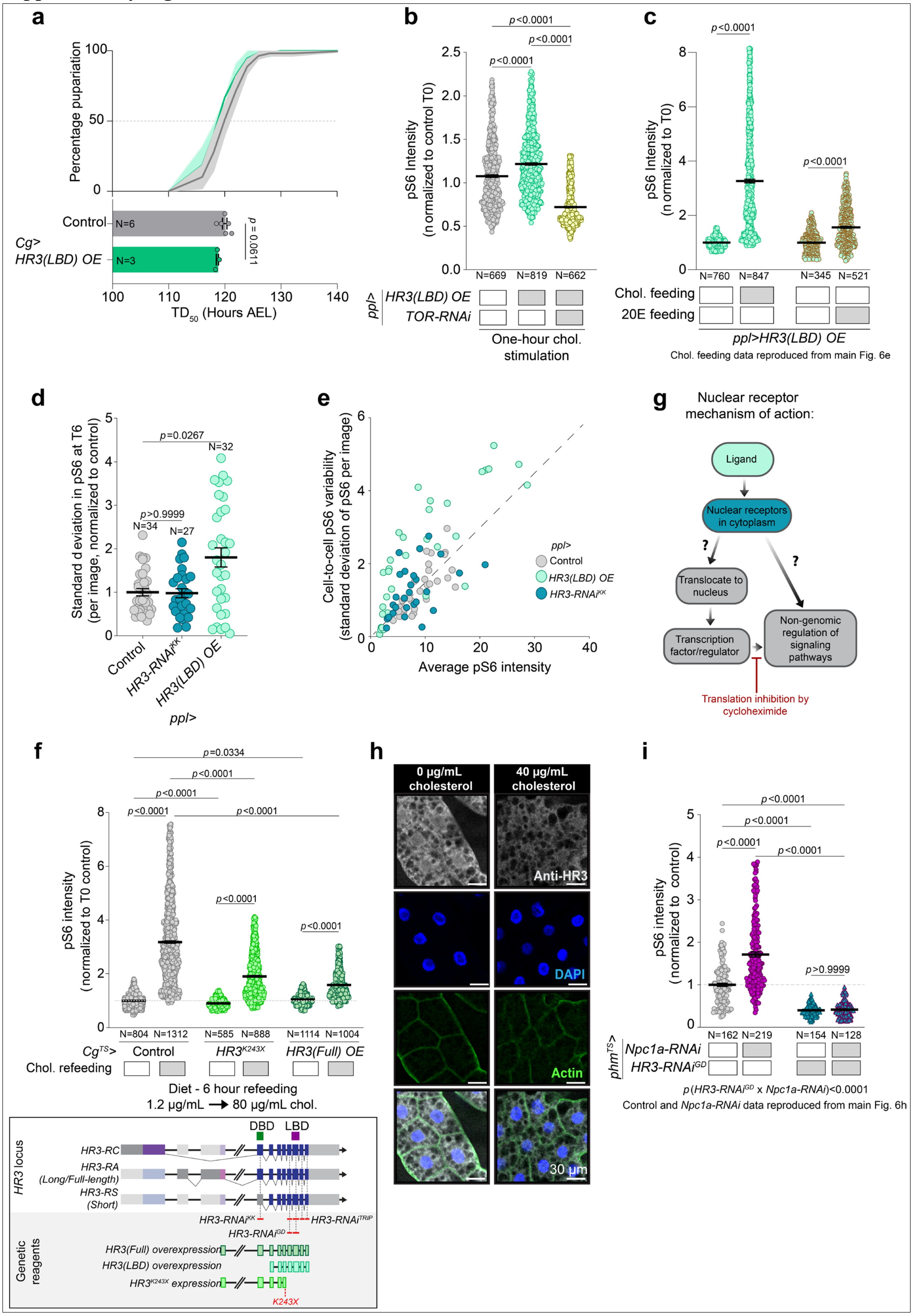

Supplementary Figure 6 (previous page). **a**, Timing of pupariation in control larvae and larvae overexpressing *HR3(LBD)* in the fat body, driven by *Cg*>. **b**, Fat-body pS6 response to 1-hour cholesterol refeeding (80 µg/mL) in controls and animals overexpressing HR3(LBD) in the fat body using *ppl*>, with and without simultaneous *TOR* knockdown, following 10-hour feeding with low-cholesterol (1.2 µg/mL) medium. **c**, Phospho-S6 responses in fat-body cells of larvae overexpressing HR3(LBD) in the fat body (*ppl*>) to feeding with cholesterol (80 µg/mL) or 20-hydroxyecdysone (20E, 250 µg/mL with 1.2 µg/mL cholesterol) for 6 hours following 10-hour feeding on low-cholesterol (1.2 µg/mL) medium. **d**, Quantification of cell-to-cell variation in pS6 intensity. **e**, Cell-to-cell variability in pS6 signal in fat-body tissue fragments of control larvae and animals either expressing *HR3* knockdown or overexpressing HR3(LBD) in the fat-body (*ppl*>) after 6 hours' cholesterol re-feeding (80 µg/mL) following 10-hour low-cholesterol feeding (1.2 µg/mL). **f**, Top: Cholesterol-induced TOR activation in animals overexpressing full-length *HR3* or the $HR3^{K243X}$ truncation allele in the fat body (Cg^{TS}>). Fat-body pS6 levels after 6 hours of cholesterol refeeding (80 µg/mL) following 10-hour feeding on 1.2 µg/mL cholesterol (conditions as in Supplementary Fig. 1e). Bottom: A schematic overview of the three constructs (full-length *HR3*, *HR3(LBD)*, and $HR3^{K243X}$) is shown below the plot, including targeted regions of the different HR3-RNAi lines. **g**, Illustration of two potential mechanisms of TOR regulation by nuclear receptors such as HR3, illustrating the impetus for the *ex-vivo* experiments. Treatment with cycloheximide inhibits one of the two routes by inhibiting translation; thus the fraction of cholesterol-induced pS6 increase remaining with cycloheximide treatment is due to non-genomic effects on the TOR signaling pathway. **h**, Representative immunostain against HR3 in larval fat-body tissue after cholesterol starvation (6 hours) and after cholesterol re-feeding (0 µg/mL for 10 hours). Scale bar is 30 µm. One tissue per condition was imaged out of at least 15 on the slide. **i**, pS6 staining in the prothoracic gland with *Npc1a-RNAi* alone and in combination with *HR3* knockdown. Statistics: Data are plotted in (a-d,f,i) as means with SEM, and *p*-values were calculated using Kruskal-Wallis ANOVA (two-sided) with Dunn's multiple comparisons and two-way ANOVA test for interactions. Data are normalized to Cg^{TS}> or phm^{TS}> driver control. (c) Each point represents the brightness and variation within one piece of fat-body tissue, such as those in panel. (d) Each data point reflects the standard deviation among all the single-cell pS6 measurements in a single analyzed tissue fragment. Source data are provided in the Source Data file.

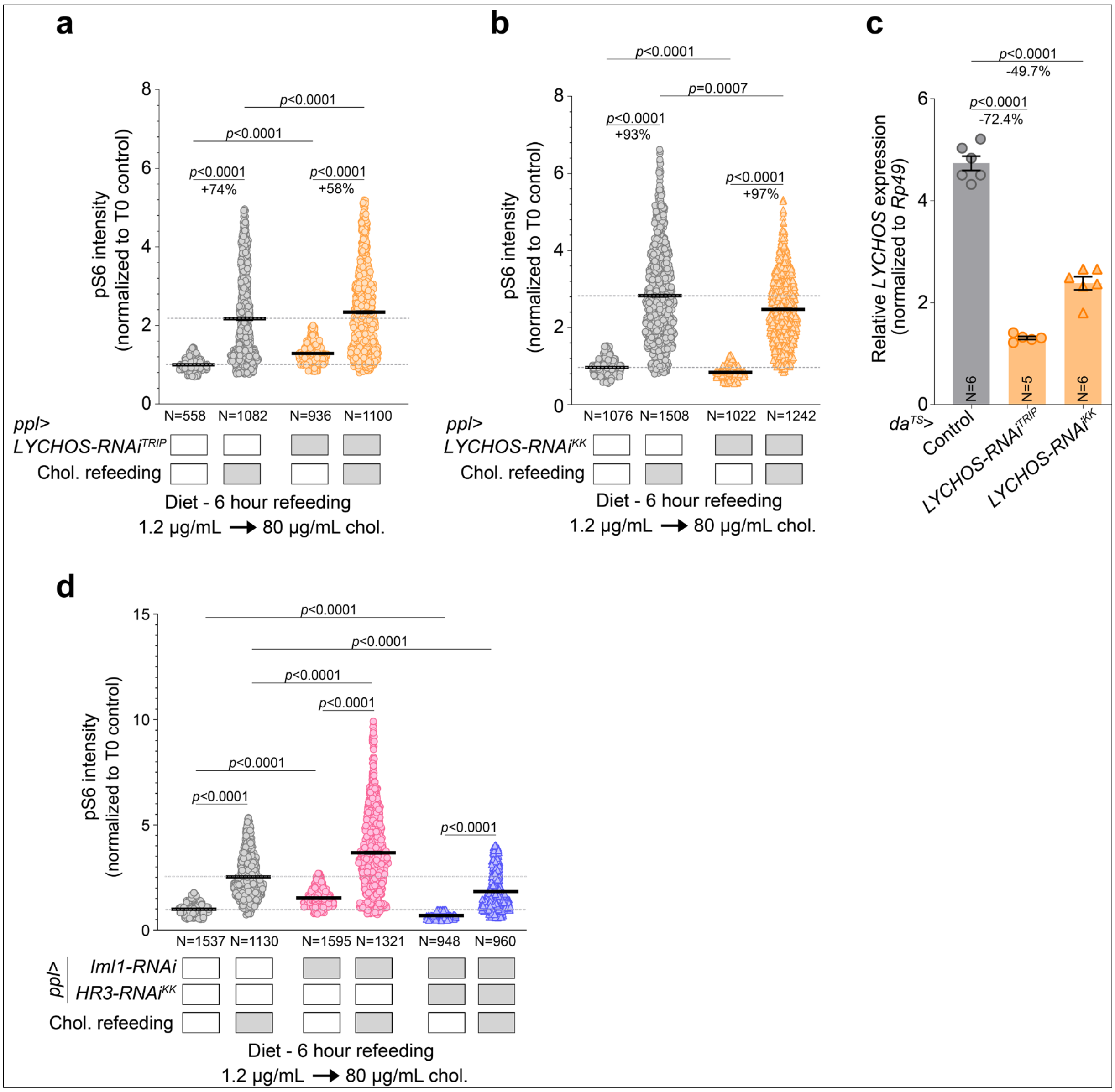

Supplementary Figure 7. a, Quantification of pS6 levels in larval fat-body cells of controls and animals expressing *LYCHOS* (*anchor*) RNAi (TRiP line) in the fat body (*ppl*>) following cholesterol refeeding. Larvae were raised on standard diet until 84 hours after egg laying (AEL), transferred to synthetic food containing 1.2 µg/mL cholesterol for 10 hours, and then re-fed for 6 hours on synthetic food containing either 1.2 µg/mL or 80 µg/mL cholesterol (conditions as in Supplementary Fig. 1a). Values are normalized to the T0 control condition. **b**, qPCR quantification of *LYCHOS* transcript levels in whole larvae of controls and animals expressing *LYCHOS* RNAi (TRiP or KK lines) using *da-GAL4; Tub-GAL80*^{TS} (*da*^{TS}>). Larvae were raised at 18 °C to maintain GAL80^{TS}-mediated GAL4 inhibition. At 120 hours AEL, larvae were shifted to 29 °C to inactivate GAL80^{TS} and induce RNAi expression during late larval stages. Animals were collected 24 hours after induction for RNA extraction and qPCR. **c**, Quantification of pS6 levels in larval fat-body cells of controls and animals expressing an independent *LYCHOS* RNAi line (KK) in the fat body (*ppl*>), following cholesterol refeeding. Larvae were treated as in (a). Values are normalized to the T0 control condition. **d**, Quantification of pS6 integrated intensity/area in larval fat-body cells of controls and animals expressing *Iml1* RNAi, *HR3* RNAi, or both in the fat body (*ppl*>), following cholesterol refeeding. Larvae were treated as in (a). Values are normalized to the T0 control condition. **Statistics**: In (a, c, d), each point represents the cytoplasmic pS6 intensity of a single cell; N values are shown below each condition. Data are shown as mean ± SEM. *p*-values in (a,c) were calculated by Kruskal–Wallis ANOVA (two-sided) with Dunn's multiple comparisons, and those in (b) were calculated by two-sided ordinary one-way ANOVA with Dunnett's multiple comparisons. Source data are provided in the Source Data file.

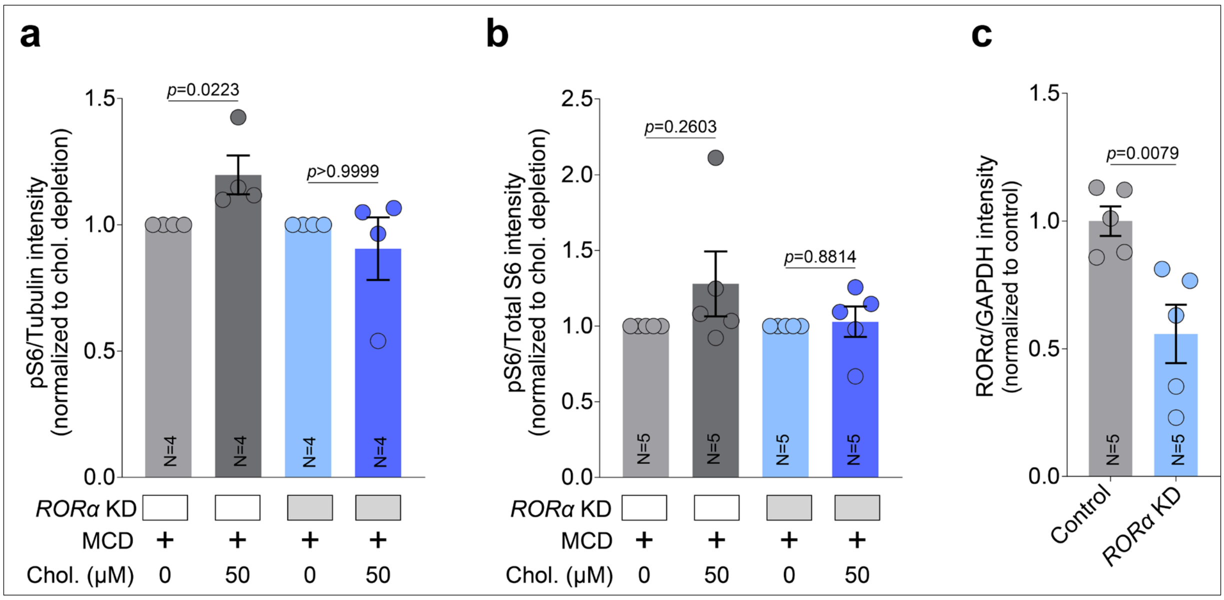

Supplementary Figure 8. a, Quantification of pS6 normalized to α-Tubulin in control and *RORα* knockdown (KD) Karpas 707H cells subjected to cholesterol depletion and replenishment. Values are normalized to the cholesterol-depleted condition for each genotype. **b**, Quantification of pS6 normalized to total S6 intensity in the same samples as in (a). **c**, Quantification of RORα protein levels normalized to GAPDH in control and *RORα* KD cells. Data represent mean ± SEM. Statistical significance was assessed using unpaired two-sided *t*-tests. Source data are provided in the Source Data file.

Supplementary Table 1. Fly lines and reagents used.

REAGENT or RESOURCE	SOURCE	IDENTIFER
**Antibodies**		
Anti-GFP, mouse clone 3E6	ThermoFisher	A11120<br>RRID AB_221568
Anti-guinea pig IgG, Alexa Fluor 488 conjugate	ThermoFisher	A21450<br>RRID AB_2735091
Anti-histone H3, rabbit	Abcam	1791; No RRID
Anti-HR3, rabbit	Gift from J. Montagne (Friedrich Miescher Institute for Biomedical Research). (Montagne *et al.*[3])	n/a
Anti-mouse IgG, Alexa Fluor 488 conjugate	ThermoFisher	A32723<br>RRID AB_2633275
Anti-phospho-S6 (*Drosophila*)	Romero-Pozuelo *et al.*[4]; Texada *et al.*[5]	n/a
Anti-phospho-S6 (Human)	Cell Signaling	4857
Anti-phospho-S6K (*Drosophila*, pT398), rabbit	Cell Signaling	9209S
Anti-rabbit IgG, Alexa Fluor 488 conjugate	ThermoFisher	A32731<br>RRID AB_2633280
Anti-rabbit IgG, Alexa Fluor 555 conjugate	ThermoFisher	A32732<br>RRID AB_2633281
Anti-rabbit IgG, Alexa Fluor 647 conjugate	ThermoFisher	#A32733<br>RRID AB_2633282
Anti-RORα (human) Rabbit mAB (E6G51)	Cell Signaling	34639S
Anti-S6 – western blot	Cell Signaling	2317
Anti-Shroud, guinea pig	Gift from R. Niwa (University of Tsukuba). (Shimada-Niwa and Niwa[6])	n/a
Anti-α-Tubulin, mouse	Developmental Studies Hybridoma Bank	AA4.3
Goat anti-mouse IgG (H+L) peroxidase conjugate	Jackson ImmunoResearch	115-035-003
Goat anti-rabbit IgG (H+L) peroxidase conjugate	Jackson ImmunoResearch	111-035-003
Goat anti-rabbit IgG (H+L) peroxidase Conjugate	ThermoFisher	31466
IRDye 680RD-labeled goat anti-mouse	Li-COR	925-68070
IRDye 800CW goat anti-rabbit	Li-COR	925-32210
Anti-GAPDH, rabbit	Cell Signaling	2118L
**Chemicals, peptides, and recombinant proteins**		
20-hydroxyecdysone (20E, ecdysone)	Sigma-Aldrich	H5142
2x Laemmli sample buffer	Bio-Rad	1610737
Agar, bacteriological grade	VWR	J637-1KG
Benzonase	Sigma-Aldrich	G9422
β-Glycerophosphate disodium salt	Sigma-Aldrich	154808-51-0
Bicinchoninic acid solution	Sigma-Aldrich	B9643-1L
Biotin	Sigma-Aldrich	B4501; CAS 58-85-5
Bovine serum albumin (protease-free, lyophilized)	BioWest	P6155-1KG
Casein	Sigma-Aldrich	C5679; CAS 9000-71-9
Chameleon Duo pre-stained protein ladder	Li-COR	928-60000
DAPI	ThermoFisher	62248; CAS 28718-90-3
Chloroform	Carlo Erba Reagents	438612
Cholesterol	Sigma-Aldrich	C3045; CAS 57-88-5
Cholesterol	Steraloids	C6807-000
Cholesterol sulfate	Steraloids	
Choline chloride	Sigma-Aldrich	C1879; CAS 67-48-1
Copper(II) sulfate solution	Sigma-Aldrich	C2284-25ML
Cyloheximide	Sigma-Aldrich	239765
7-**D**ehydrocholesterol	Steraloids	C3000-000
DNase	Macherey-Nagel	740955.250

D-Pantothenic acid, hemicalcium salt	Sigma-Aldrich	P5155; CAS 137-08-6
Farnesol	Sigma	93547
Farnesyl acetate	Sigma	340480
Fetal bovine serum (FBS, Qualified, HI)	Gibco	10500-064
Fetal bovine serum	Wisent	090-110
Folic acid	Sigma-Aldrich	F8758; CAS 59-30-3
Geraniol	Sigma	163333
GSK157369x	GlaxoSmithKline	
Imidazole	Sigma	I2399
Inosine	Sigma-Aldrich	I4125; CAS 58-63-9
Intercept blocking buffer	Li-COR	927-70001
IPTG	Bioshop	IPT002
Lipofectamine 2000	Invitrogen	11668-030
Lipoprotein deficient serum from fetal calf (Lipid-depleted serum, LDS)	Sigma-Aldrich	S5394-50mL
Methoprene	Sigma	33375
Methyl-β-Cyclodextrin (MCD)	Sigma-Aldrich	C4555
M3 medium (Shields and Sang)	Sigma	S3652
Nicotinic acid	Sigma-Aldrich	N0761; CAS 59-67-6
NutriFly medium	Genesee Scientific	‘‘Bloomington formula,’’ 66-112
N,O-BSTFA	Sigma	155195
Opti-MEM (1X) reduced-serum medium	Gibco	31985-062
Penicillin-Streptomycin (Pen-Strep)	Gibco	15140-122
Phalloidin, Alexa Fluor 488 conjugate	ThermoFisher	A12379
Phalloidin, Alexa Fluor 647 conjugate	ThermoFisher	A22287
PhosStop – phosphatase inhibitor tablets	Roche	PHOSS-RO
PMSF	Sigma	10837091001
Poly-lysine	Sigma-Aldrich	P8920-100ML
ProLong Glass Antifade Mountant	Life Technologies	P36984
Propanediol	Sigma	12279
Pyridoxine	Sigma-Aldrich	P6280; CAS 58-56-0
Riboflavin	Sigma-Aldrich	R4500; CAS 83-88-5
Roche Complete Protease Inhibitor cocktail with EDTA (tablets)	Roche	11836153001
Schneider’s *Drosophila* Medium (1x)	Gibco	21720
Skim milk powder	Millipore	70166-500G
Squalene	Sigma	S3626
StableCell RPMI-1640	Sigma-Aldrich	R2405-500ML
Stearic acid	Sigma	175366
Superdex 16/60 resin	GE Healthcare	n/a
TCEP	Sigma	C4706
Tegosept (Methyl 4-hydroxybenzoate)	VWR	2505.293
Thiamine	Sigma-Aldrich	T1270; CAS 67-03-8
Trypsin-EDTA (0.25%)	Gibco	25200056
Tween20	Sigma-Aldrich	P9416-100ML
Uridine	Sigma-Aldrich	U3003; CAS 58-96-8
VectaShield Antifade Mounting Medium with DAPI	Vector Laboratories	H-1200
**Critical commercial assays and kits**		
RNeasy Mini Kit	Qiagen	74104
Steady-Glo Luciferase Assay System	Promega	E2510
Glo Lysis Buffer	Promega	E2661
Trans-Blot Turbo Midi 0.2 µm Nitrocellulose Transfer Packs	Bio-Rad	1704159
ECL Prime Western Blotting Detection Reagents	Amersham	RPN2232
10% Mini-PROTEAN TGX precast polyacrylamide gel	Bio-Rad	4561034
12% Mini-PROTEAN TGX precast polyacrylamide gel	Bio-Rad	4561044
4-20% Mini-PROTEAN TGX Precast polyacrylamide gel	Bio-Rad	4561094
SecureSeal imaging spacers	Grace Bio-labs	GBL654006-100EA

**Deposited Data**		
Transcriptomic data	NCBI GEO	Accession number GSE270221
Phosphoproteomic data	ProteomeXchange Consortium (PRIDE)	Dataset identifier PXD074014
**Experimental models: cell lines**		
Karpas-707H cells	University of Cambridge (Karpas *et al.*[7])	https://www.enterprise.cam.ac.uk/reagents/karpas-707h-human-myeloma-cell-line/
Hi5 insect cells (*Trichoplusia ni*)	ThermoFisher	#B85502
**Experimental models: *Drosophila* stocks**		
Cg-GAL4	Bloomington *Drosophila* Stock Center (BDSC) (Asha *et al.*[8])	7011
Heat-shock-GAL4(DBD)::HR3(LBD);UAS-GFP	BDSC (Palanker *et al.*[9])	28868
phm-GAL4 TubGAL80^{TS}	n/a	Made from BDSC 7018
phm-GAL4; Tub-GAL80^{TS}	McGuire *et al.*[10]	Made from BDSC 7108 and phm$^{\#22}$-GAL4 (Gift from M. O'Connor, University of Minnesota)
pumpless (ppl)-GAL4	BDSC (Zinke *et al.*[11])	58768
Tub-GAL80^{TS}; da-GAL4	n/a	Made from BDSC 7108 and 55850
Tub-GAL80^{TS}; Tub-GAL4	n/a	86328
UAS-anchor-RNAi^{KK} (LYCHOS)	VDRC	105969
UAS-anchor-RNAi^{TRIP} (LYCHOS)	BDSC	51463
UAS-dib-RNAi	VDRC	101117
UAS-EcR-RNAi	BDSC (Ni *et al.*[12])	37058 and 37059
UAS-HR3(LBD) OE	Gift from J. Montagne. (Montagne *et al.*[3])	DHR3-RS
UAS-HR3-RNAi^{KK}	Vienna *Drosophila* Resource Center (VDRC). (Dietzl *et al.*[13])	106837
UAS-HR3(Full) OE	This study	
UAS-HR3^{K243X}	This study	
UAS-HR3-RNAi^{GD}	VDRC	12044
UAS-HR3-RNAi^{TRiP}	BDSC	27253
UAS-Iml1-RNAi	BDSC	57492
UAS-Npc1a-RNAi^{KK}	VDRC	105405
UAS-phm-RNAi	VDRC	108359
UAS-RagA-B-RNAi	BDSC	34590
UAS-RagC-D-RNAi	BDSC	32342
UAS-S6K-RNAi	VDRC	104369
UAS-S6K^{STED}	BDSC	6913
UAS-TOR-RNAi	BDSC	34639
UAS-torso-RNAi		33627
unk-FireflyLuciferase (B76)	Gift from A. Teleman (Tiebe *et al.*[14])	n/a
w^{1118}	VDRC	60000
**Oligonucleotides for knockdown**		
Human *RORα* siGenome SMARTpool	Horizon - Dharmacon	M-003440-01-0005
**Quantitative RT-PCR Oligos**		
anchor (*LYCHOS*)	F: GGGGCGTATCCATGAACAACT	R: TTGAAGCGTCCGGCAATGTAA
Rp49	F: AGTATCTGATGCCCAACATCG	R: CAATCTCCTTGCGCTTCTTG
**Software and algorithms**		
Boltz-2	n/a	Wohlwend *et al.*[15]; Passaro *et al.*[16]
CellProfiler 3.1.9	http://cellprofiler.org/	McQuin *et al.*[17]
FIJI (ImageJ) 1.54p	http://fiji.sc/	Schneider *et al.*[18]
Mascot v 2.2.04	Matrix Science Ltd.	n/a

Mass Lynx 4.0	Micromass	n/a
MS Workstation	Varian	n/a
PANTHER – Classification system – version 18.0	pantherdb.org	Thomas *et al.*[19]
Prism 10	GraphPad	n/a
Proteome Discoverer v2.5.0.400	ThermoFisher Scientific	n/a
R, 4.5.0	https://www.r-project.org/	n/a
SRplot	SRplot - Free online GO, Pathway bubble plot (bioinformatics.com.cn)	Tang *et al.*[20]
STRING version 12.0	STRING: functional protein association networks (string-db.org)	Szklarczyk *et al.*[21]
Xcalibur v3.0	ThermoFisher Scientific	n/a
ZEN 3.5 (ZEN lite)	Zeiss	n/a
**Other**		
AKTA FPLC apparatus	GE Healthcare	n/a
3900 gas chromatograph	Varian	n/a
Saturn 2100T ion-trap mass spectrometer	Varian	n/a
Quadropole time-of-flight mass spectrometer	Micromass	n/a
Fused silica column	Varian	CP8944
Tandem mass tags 18-plex	ThermoFisher Scientific	A52045
EASY nanoLC	ThermoFisher Scientific	n/a
Fusion Lumos Tribrid mass spectrometer	ThermoFisher Scientific	n/a
Orbitrap Eclipse Tribrid mass spectrometer	ThermoFisher Scientific	n/a

Supplementary Table 2. DNA sequences of *HR3* constructs.

1. DNA sequence of *HR3*$^{Full\text{-}length}$

EcoRI Kozak Hr3-PA Predicted DBD K243 Predicted LBD XbaI

GAATTCGCCACCATGGAATACCCCTACGATGTGCCCGATTACGCCGCTGGCGAGTACCCGTATGATGTGCCAGAC
TATGCTGCCGGCGAGTATCCATACGACGTGCCGGATTATGCAGCCGGCATTAGCGTGCTGAGCGGCATGTAT**ACC**
CAGCGCATGTTCGATATGTGGTCCAGCGTGACCAGCAAGCTGGAAGCCCACGCCAACAATCTGGGCCAGAGCAAT
GTGCAAAGCCCAGCCGGCCAGAACAATAGCAGCGGCAGTATCAAGGCCCAGATCGAGATCATCCCCTGCAAAGTG
TGCGGCGATAAGAGCAGCGGAGTGCACTACGGCGTGATCACATGCGAGGGCTGCAAGGGATTCTTTCGCCGTAGC
CAGAGCAGCGTGGTCAACTATCAGTGCCCGCGCAACAAGCAGTGCGTGGTGGATCGCGTGAACCGCAATCGCTGC
CAGTATTGCCGTCTGCAGAAGTGCCTGAAGCTGGGCATGAGTCGTGATGCCGTGAAGTTCGGCCGCATGAGCAAG
AAACAGCGCGAGAAGGTGGAAGATGAAGTGCGCTTCCACCGCGCTCAGATGCGTGCTCAAAGTGATGCCGCTCCG
GATAGCAGCGTGTACGATACCCAAACGCCGAGCAGCAGCGATCAGCTGCACCACAACAACTACAACAGCTACAGC
GGCGGCTACAGCAACAACGAAGTCGGCTACGGAAGCCCCTACGGATATAGCGCTAGTGTGACCCCGCAGCAGACC
ATGCAGTACGATATCAGCGCCGATTACGTGGACAGCACCACCTATGAGCCACGCAGCACCATCATCGATCCCGAG
TTCATCAGCCACGCCGATGGCGATATCAACGATGTGCTG**ATCAAGACCCTGGCCGAGGCGCACGCCAATACCAAC**
ACAAAGTTGGAGGCCGTGCACGACATGTTCCGCAAGCAGCCAGATGTGTCCCGCATCCTGTACTACAAGAACCTG
GGCCAAGAGGAACTGTGGCTGGATTGCGCCGAGAAGCTGACCCAGATGATCCAGAACATCATCGAGTTCGCCAAG
CTGATCCCCGGCTTCATGCGTCTGAGCCAGGATGATCAGATCCTGCTGCTGAAAACCGGCAGCTTCGAGCTGGCC
ATTGTGCGCATGAGCCGTCTGCTGGATCTGTCCCAAAACGCCGTGCTGTACGGCGACGTGATGCTGCCACAAGAG
GCCTTCTACACCAGCGATAGCGAAGAGATGCGCCTGGTGTCGCGCATTTTCCAGACCGCCAAGAGCATTGCCGAG
CTGAAGCTGACGGAAACAGAGCTGGCCCTGTACCAGAGTCTGGTGCTGTTGTGGCCAGAGCGCAATGGCGTGCGT
GGCAATACCGAAATCCAGCGCCTGTTCAACCTGAGCATGAACGCCATTCGCCAAGAGTTGGAGACAAATCACGCC
CCGTTGAAGGGCGACGTCACCGTGTTGGATACCCTGCTGAACAACATCCCCAACTTCCGCGATATCTCCATCCTG
CACATGGAAAGCCTGAGCAAGTTCAAGCTGCAGCACCCCAACGTGGTGTTCCCCGCCTTGTACAAAGAGCTGTTC
AGCATCGATAGCCAGCAGGACCTGACCTAATCTAGA

2. DNA sequence of *HR3*$^{DBD\text{-}only}$

EcoRI Kozak Hr3-PA Predicted DBD K243X (AAG→TAA) Predicted LBD XbaI

GAATTCGCCACCATGGAATACCCCTACGATGTGCCCGATTACGCCGCTGGCGAGTACCCGTATGATGTGCCAGAC
TATGCTGCCGGCGAGTATCCATACGACGTGCCGGATTATGCAGCCGGCATTAGCGTGCTGAGCGGCATGTAT**ACC**
CAGCGCATGTTCGATATGTGGTCCAGCGTGACCAGCAAGCTGGAAGCCCACGCCAACAATCTGGGCCAGAGCAAT
GTGCAAAGCCCAGCCGGCCAGAACAATAGCAGCGGCAGTATCAAGGCCCAGATCGAGATCATCCCCTGCAAAGTG
TGCGGCGATAAGAGCAGCGGAGTGCACTACGGCGTGATCACATGCGAGGGCTGCAAGGGATTCTTTCGCCGTAGC
CAGAGCAGCGTGGTCAACTATCAGTGCCCGCGCAACAAGCAGTGCGTGGTGGATCGCGTGAACCGCAATCGCTGC
CAGTATTGCCGTCTGCAGAAGTGCCTGAAGCTGGGCATGAGTCGTGATGCCGTGAAGTTCGGCCGCATGAGCAAG
AAACAGCGCGAGAAGGTGGAAGATGAAGTGCGCTTCCACCGCGCTCAGATGCGTGCTCAAAGTGATGCCGCTCCG
GATAGCAGCGTGTACGATACCCAAACGCCGAGCAGCAGCGATCAGCTGCACCACAACAACTACAACAGCTACAGC
GGCGGCTACAGCAACAACGAAGTCGGCTACGGAAGCCCCTACGGATATAGCGCTAGTGTGACCCCGCAGCAGACC
ATGCAGTACGATATCAGCGCCGATTACGTGGACAGCACCACCTATGAGCCACGCAGCACCATCATCGATCCCGAG
TTCATCAGCCACGCCGATGGCGATATCAACGATGTGCTG**ATC**TAATCTAGA

Supplementary References

1 Kallen, J., Schlaeppi, J. M., Bitsch, F., Delhon, I. & Fournier, B. Crystal structure of the human RORalpha Ligand binding domain in complex with cholesterol sulfate at 2.2 A. *J Biol Chem* **279**, 14033–14038 (2004). https://doi.org/10.1074/jbc.M400302200

2 Kallen, J. A. *et al.* X-ray structure of the hRORalpha LBD at 1.63 A: structural and functional data that cholesterol or a cholesterol derivative is the natural ligand of RORalpha. *Structure* **10**, 1697–1707 (2002).

3 Montagne, J. *et al.* The nuclear receptor DHR3 modulates dS6 kinase-dependent growth in Drosophila. *PLoS Genet* **6**, e1000937 (2010). https://doi.org/10.1371/journal.pgen.1000937

4 Romero-Pozuelo, J., Demetriades, C., Schroeder, P. & Teleman, A. A. CycD/Cdk4 and Discontinuities in Dpp Signaling Activate TORC1 in the Drosophila Wing Disc. *Dev Cell* **42**, 376–387 e375 (2017). https://doi.org/10.1016/j.devcel.2017.07.019

5 Texada, M. J. *et al.* A fat-tissue sensor couples growth to oxygen availability by remotely controlling insulin secretion. *Nat Commun* **10**, 1955 (2019). https://doi.org/10.1038/s41467-019-09943-y

6 Shimada-Niwa, Y. & Niwa, R. Serotonergic neurons respond to nutrients and regulate the timing of steroid hormone biosynthesis in Drosophila. *Nat Commun* **5**, 5778 (2014). https://doi.org/10.1038/ncomms6778

7 Karpas, A., Dremucheva, A. & Czepulkowski, B. H. A human myeloma cell line suitable for the generation of human monoclonal antibodies. *Proc Natl Acad Sci U S A* **98**, 1799–1804 (2001). https://doi.org/10.1073/pnas.98.4.1799

8 Asha, H. *et al.* Analysis of Ras-induced overproliferation in Drosophila hemocytes. *Genetics* **163**, 203–215 (2003). https://doi.org/10.1093/genetics/163.1.203

9 Palanker, L. *et al.* Dynamic regulation of Drosophila nuclear receptor activity in vivo. *Development* **133**, 3549–3562 (2006). https://doi.org/10.1242/dev.02512

10 McGuire, S. E., Mao, Z. & Davis, R. L. Spatiotemporal gene expression targeting with the TARGET and gene-switch systems in Drosophila. *Sci STKE* **2004**, pl6 (2004). https://doi.org/10.1126/stke.2202004pl6

11 Zinke, I., Kirchner, C., Chao, L. C., Tetzlaff, M. T. & Pankratz, M. J. Suppression of food intake and growth by amino acids in Drosophila: the role of pumpless, a fat body expressed gene with homology to vertebrate glycine cleavage system. *Development* **126**, 5275–5284 (1999). https://doi.org/10.1242/dev.126.23.5275

12 Ni, J. Q. *et al.* A Drosophila resource of transgenic RNAi lines for neurogenetics. *Genetics* **182**, 1089–1100 (2009). https://doi.org/10.1534/genetics.109.103630

13 Dietzl, G. *et al.* A genome-wide transgenic RNAi library for conditional gene inactivation in Drosophila. *Nature* **448**, 151–156 (2007). https://doi.org/10.1038/nature05954

14 Tiebe, M. *et al.* REPTOR and REPTOR-BP Regulate Organismal Metabolism and Transcription Downstream of TORC1. *Dev Cell* **33**, 272–284 (2015). https://doi.org/10.1016/j.devcel.2015.03.013

15 Wohlwend, J. *et al.* Boltz-1 Democratizing Biomolecular Interaction Modeling. *bioRxiv* (2025). https://doi.org/10.1101/2024.11.19.624167

16 Passaro, S. *et al.* Boltz-2: Towards Accurate and Efficient Binding Affinity Prediction. *bioRxiv* (2025). https://doi.org/10.1101/2025.06.14.659707
17 McQuin, C. *et al.* CellProfiler 3.0: Next-generation image processing for biology. *PLoS Biol* **16**, e2005970 (2018). https://doi.org/10.1371/journal.pbio.2005970
18 Schneider, C. A., Rasband, W. S. & Eliceiri, K. W. NIH Image to ImageJ: 25 years of image analysis. *Nat Methods* **9**, 671–675 (2012). https://doi.org/10.1038/nmeth.2089
19 Thomas, P. D. *et al.* PANTHER: Making genome-scale phylogenetics accessible to all. *Protein Sci* **31**, 8–22 (2022). https://doi.org/10.1002/pro.4218
20 Tang, D. *et al.* SRplot: A free online platform for data visualization and graphing. *PLoS One* **18**, e0294236 (2023). https://doi.org/10.1371/journal.pone.0294236
21 Szklarczyk, D. *et al.* The STRING database in 2023: protein-protein association networks and functional enrichment analyses for any sequenced genome of interest. *Nucleic Acids Res* **51**, D638–D646 (2023). https://doi.org/10.1093/nar/gkac1000
