# Supplementary material for: HR3/RORα-mediated cholesterol sensing regulates TOR signaling
Source: Nat Commun. 2026 Mar 30;17:4609. doi: 10.1038/s41467-026-71059-x (PMC13199448; doi:10.1038/s41467-026-71059-x)
Supplement: Supplementary file 3 — Description of Additional Supplementary Files [file 41467_2026_71059_MOESM3_ESM.pdf]

Description of Additional Supplementary Files

File name: Supplementary Data 1
Description: Phosphoproteomic changes detected following *Drosophila* larval cholesterol refeeding. Related to Figure 2d, 2e, 2f, and 2g and to Supplementary Figure 2a, 2b, 2c, and 2d. *P*-values for differences between samples were computed using two-sided ANOVA followed by Benjamini-Hochberg FDR estimation.

File name: Supplementary Data 2
Description: Phosphoproteomic changes detected over time after larval refeeding with protein. Related to Figure 2d, 2e, 2f, and 2h and to Supplementary Figure 2a, 3a, and 3b. *P*-values for differences were computed using two-sided ANOVA with Benjamini-Hochberg FDR estimation.

File name: Supplementary Data 3
Description: Transcriptomic changes in controls, *HR3* KD, and *TOR* KD, after cholesterol depletion and following cholesterol refeeding. Related to Fig. 5d and 5e. *P*-values for differential gene expression were computed by Wade test and adjusted for multiple comparisons using the Benjamini-Hochberg FDR procedure; both numbers are given in the file.

File name: Supplementary Data 4
Description: Phosphoproteomic changes detected in control, *HR3*-knockdown, and *TOR*-knockdown larvae following cholesterol refeeding. Related to Fig. 5f, 5g, 5h, 5i, 5j, and 5k and to Supplementary Figure 5f. *P*-values for differences were computed using two-sided ANOVA with Benjamini-Hochberg FDR estimation.

File name: Supplementary Data 5
Description: Phosphoproteomic changes in Karpas 707H cells after cholesterol stimulation. Related to Fig. 7d, 7e, 7g, and 7h. *P*-values for differences were computed using two-sided ANOVA with Benjamini-Hochberg FDR estimation.
