# Supplementary material for: HR3/RORα-mediated cholesterol sensing regulates TOR signaling
Source: Nat Commun. 2026 Mar 30;17:4609. doi: 10.1038/s41467-026-71059-x (PMC13199448; doi:10.1038/s41467-026-71059-x)
Supplement: Supplementary file 9 — Reporting Summary [file 41467_2026_71059_MOESM9_ESM.pdf]

nature portfolio

Corresponding author(s): Rewitz K

Last updated by author(s): Dec 18, 2025

Reporting Summary

Nature Portfolio wishes to improve the reproducibility of the work that we publish. This form provides structure for consistency and transparency in reporting. For further information on Nature Portfolio policies, see our Editorial Policies and the Editorial Policy Checklist.

Statistics

For all statistical analyses, confirm that the following items are present in the figure legend, table legend, main text, or Methods section.

n/a	Confirmed	
☐	☒	The exact sample size (*n*) for each experimental group/condition, given as a discrete number and unit of measurement
☐	☒	A statement on whether measurements were taken from distinct samples or whether the same sample was measured repeatedly
☐	☒	The statistical test(s) used AND whether they are one- or two-sided<br>*Only common tests should be described solely by name; describe more complex techniques in the Methods section.*
☒	☐	A description of all covariates tested
☐	☒	A description of any assumptions or corrections, such as tests of normality and adjustment for multiple comparisons
☐	☒	A full description of the statistical parameters including central tendency (e.g. means) or other basic estimates (e.g. regression coefficient) AND variation (e.g. standard deviation) or associated estimates of uncertainty (e.g. confidence intervals)
☐	☒	For null hypothesis testing, the test statistic (e.g. *F*, *t*, *r*) with confidence intervals, effect sizes, degrees of freedom and *P* value noted<br>*Give P values as exact values whenever suitable.*
☒	☐	For Bayesian analysis, information on the choice of priors and Markov chain Monte Carlo settings
☒	☐	For hierarchical and complex designs, identification of the appropriate level for tests and full reporting of outcomes
☒	☐	Estimates of effect sizes (e.g. Cohen's *d*, Pearson's *r*), indicating how they were calculated

Our web collection on statistics for biologists contains articles on many of the points above.

Software and code

Policy information about availability of computer code

Data collection	* Zeiss Zen Blue v3.1 for image acquisition<br>* Xcalibur v3.0 (ThermoFisher Scientific) for raw phosphoproteomics data
Data analysis	* FIJI/ImageJ (NIH), vl.53, for image analysis (Schneider CA, Rasband WS, and Eliceiri KW, 2012. Nat Methods 9, 671-675)<br>* Prism, v10 (GraphPad) for numerical analysis and presentation<br>* Proteome Discoverer version 2.5.0.400 (ThermoFisher Scientific) for phosphoproteomic analysis<br>* SEQUEST HT for phosphoproteomic analysis<br>* Percolator for phosphoproteomic analysis<br>* CellProfiler, v. 3.1.9, http://cellprofiler.org/ (McQuin C et al., 2018. PLoS Biol 16, e2005970)<br>* PANTHER Classification system, version 18.0, http://pantherdb.org/ (Thomas PD et al., 2022. Protein Sci 31, 8-22)<br>* STRING functional protein association networks, version 12.0 (stringdb.org) Szklarczyk D et al., 2023. Nucleic Acids Res 51, D638-D646<br>* Xcalibur v3.0 (ThermoFisher Scientific)<br>* MS Workstation (Varian)<br>* Mascot v 2.2.04 (Matrix Science Ltd)<br>* Mass Lynx 4.0 (Micromass)<br>* Boltz-2 for structural protein/ligand docking, described here: https://www.biorxiv.org/content/10.1101/2025.06.14.659707v1<br>* R, version 4.5.0

For manuscripts utilizing custom algorithms or software that are central to the research but not yet described in published literature, software must be made available to editors and reviewers. We strongly encourage code deposition in a community repository (e.g. GitHub). See the Nature Portfolio guidelines for submitting code & software for further information.

Data

Policy information about availability of data

All manuscripts must include a data availability statement. This statement should provide the following information, where applicable:

- Accession codes, unique identifiers, or web links for publicly available datasets
- A description of any restrictions on data availability
- For clinical datasets or third party data, please ensure that the statement adheres to our policy

All data generated in this study are available within this manuscript, its figures, or its supplementary files or on public databases. Source data are provided with this paper. The transcriptomic data generated for this study have been deposited in the NCBI Gene Expression Omnibus (GEO) repository under the accession code GSE270221 [https://www.ncbi.nlm.nih.gov/geo/query/acc.cgi?acc=GSE270221]. The mass-spectrometry proteomics data generated for this work have been deposited to the ProteomeXchange Consortium via the PRIDE partner repository under the dataset identifier PXD074014 [https://proteomecentral.proteomexchange.org/cgi/GetDataset?ID=PXD074014]. All data required for proper interpretation of this work is available within this manuscript or its associated Source and Supplementary Data files or has been deposited. Further data requests, such as for raw imagery, should be directed to the Lead Author.

Research involving human participants, their data, or biological material

Policy information about studies with human participants or human data. See also policy information about sex, gender (identity/presentation), and sexual orientation and race, ethnicity and racism.

Reporting on sex and gender	Karpas-707H cells [Karpas, A., Dremucheva, A., and Czepulkowski, B.H. (2001). Proc Natl Acad Sci USA 98, 1799-1804] were obtained from University of Cambridge Enterprise (https://www.enterprise.cam.ac.uk/reagents/karpas-707h-human-myeloma-cell-line/).
Reporting on race, ethnicity, or other socially relevant groupings	Information beyond the karyotype of the human cell line is not readily available.
Population characteristics	Karpas-707 cell line was established from the bone marrow of a 53 year old male with multiple myeloma:<br>Karpas, A., et al. (1982). "HUMAN PLASMACYTOMA WITH AN UNUSUAL KARYOTYPE GROWING IN VITRO AND PRODUCING LIGHT-CHAIN IMMUNOGLOBULIN." The Lancet 319(8278): 931-933.<br>https://www.cellosaurus.org/CVCL_9552
Recruitment	*Describe how participants were recruited. Outline any potential self-selection bias or other biases that may be present and how these are likely to impact results.*
Ethics oversight	No ethics approval or oversight is required for studies using the human Karpas cells. A MTA was established with University of Cambridge Enterprise for the use of the cells for this study.

Note that full information on the approval of the study protocol must also be provided in the manuscript.

Field-specific reporting

Please select the one below that is the best fit for your research. If you are not sure, read the appropriate sections before making your selection.

☒ Life sciences ☐ Behavioural & social sciences ☐ Ecological, evolutionary & environmental sciences

For a reference copy of the document with all sections, see nature.com/documents/nr-reporting-summary-flat.pdf

Life sciences study design

All studies must disclose on these points even when the disclosure is negative.

Sample size	The sample size was determined based on similar studies on Drosophila metabolism previously published in the field (doi.org:10.1038/s41467-022-28268-x, doi.org:10.1016/j.cmet.2018.09.021, doi.org:10.1038/s42255-020-0266-x, doi.org:10.1038/s42255-022-00672-z), and no specific sample-size calculations were conducted. The chosen numbers are sufficiently large to reflect typical variability while remaining manageable for experimental preparation, aligning with or exceeding the norms of published research in this area. For developmental growth, individual larvae were weighed. Luciferase, immunoblotting, protein measurements, RNA-seq assays involved around 3-5 replicates each, including multiple animals. Quantitative phosphoproteomics used 2-3 replicates with 10-15 animals in each. The image analysis incorporated multiple tissues per genotype or condition, involving 15-20 animals.
Data exclusions	No data were excluded.
Replication	Experiments producing numerical data generally contained at least 3 replicates consisting of 10-15 larvae each. Phosphoproteomics data consisted of two replicates, and transcriptomic data included five. No attempted replications were excluded.
Randomization	Animals of known genotypes or food condition but of unknown sex were randomly collected before testing

Blinding

Researchers were not blinded during the study because this is not generally done in fly studies. With limited staff with expertise in these particular studies, the person handling sample prep must usually also be the one performing the assay.

Reporting for specific materials, systems and methods

We require information from authors about some types of materials, experimental systems and methods used in many studies. Here, indicate whether each material, system or method listed is relevant to your study. If you are not sure if a list item applies to your research, read the appropriate section before selecting a response.

Materials & experimental systems

n/a	Involved in the study
☐	☒ Antibodies
☐	☒ Eukaryotic cell lines
☒	☐ Palaeontology and archaeology
☐	☒ Animals and other organisms
☒	☐ Clinical data
☒	☐ Dual use research of concern
☒	☐ Plants

Methods

n/a	Involved in the study
☒	☐ ChIP-seq
☒	☐ Flow cytometry
☒	☐ MRI-based neuroimaging

Antibodies

Antibodies used

IMMUNOSTAINING -- PRIMARIES
* Mouse monoclonal anti-GFP (clone 3E6, ThermoFisher #A11120, RRID AB_221568), 1:500
* Rabbit anti-Drosophila HR3, gift from J. Montagne (Friedrich Miescher Intitute for Biomedical Research, Basel; Montagne J et al., 2010. PLoS Genet 6, e1000937), 1:250
* Rabbit anti-Drosophila phospho-S6 peptide (RRR(phospho-S)A(phospho-S)IRE(phospho-S)K; Romero-Pozuelo J et al., 2017. Dev Cell 42, 376-387 e375; Texada MJ et al., 2019. Nature Communications 10, 1955. 10.1038/s41467-019-09943-y.), 1:500
* Guinea-pig anti-Drosophila Shroud, gift from Ryusuke Niwa (University of Tsukuba; (Shimada-Niwa Y and Niwa R , 2014. Nat Communications 5, 5778. 10.1038/ncomms6778), 1:200

IMMUNOSTAINING -- SECONDARIES
Alexa Fluor 488-conjugated goat anti-mouse (ThermoFisher #A32723, RRID AB_2633280), 1:500
Alexa Fluor 488-conjugated goat anti-rabbit (ThermoFisher #A32731, RRID AB_2633275), 1:500
Alexa Fluor 555-conjugated goat anti-rabbit (ThermoFisher #A32732 RRID AB_2633275), 1:500
Alexa Fluor 647-conjugated goat anti-rabbit (ThermoFisher #A32733, RRID AB_2633282), 1:500
Alexa Fluor 488-conjugated goat anti-guinea pig (ThermoFisher #A21450, RRID AB_2735091), 1:500

WESTERN BLOTTING -- PRIMARIES
Rabbit anti-human phospho-S6 (Cell Signaling #4857), 1:1000
Rabbit anti-histone H3 (Abcam #1791), 1:1000
Rabbit anti-human RORalpha (clone E6G51, Cell Signaling #34639S), 1:1000
Mouse anti-human S6 for Westerns (Cell Signaling #2317), 1:1000
Rabbit anti-Drosophila S6 kinase, phosphoT398 (Cell Signaling #9209S), 1:1000
Mouse anti-alpha-Tubulin (University of Iowa Developmental Studies Hybridoma Bank, #AA4.3), 1:5000
Rabbit anti-human-GAPDH (clone 14C10, Cell Signaling #2118L), 1:2500

WESTERN BLOTTING -- SECONDARIES
Goat anti-mouse IgG (H+L), peroxidase conjugate (Jackson ImmunoResearch #115-035-003), 1:10,000
Goat anti-rabbit IgG (H+L), peroxidase conjugate (Jackson ImmunoResearch #111-035-003), 1:10,000
Goat anti-rabbit IgG (H+L), peroxidase conjugate (ThermoFisher #32466), 1:10,000
Goat anti-mouse, IRDye 680RD conjugate (Li-COR #925-68070), 1:10,000
Goat anti-rabbit, IRDye 800CW conjugate (Li-COR #925-32210), 1:10,000

Validation

IMMUNOSTAINING -- PRIMARIES
* Anti-GFP (ThermoFisher #A11120) validated for staining by manufacturer: https://www.thermofisher.com/antibody/product/GFP-Antibody-clone-3E6-Monoclonal/A-11120
* Rabbit anti-Drosophila HR3 validated in Montagne J et al., 2010. PLoS Genet 6, e1000937.
* Rabbit anti-Drosophila phospho-S6 peptide validated in Romero-Pozuelo J et al., 2017. Dev Cell 42, 376-387 e375; and Texada MJ et al., 2019. Nature Communications 10, 1955. 10.1038/s41467-019-09943-y.
* Guinea-pig anti-Drosophila Shroud validated in Shimada-Niwa Y and Niwa R , 2014. Nat Communications 5, 5778. 10.1038/ncomms6778

IMMUNOSTAINING -- SECONDARIES
* AF488 goat anti-mouse(ThermoFisher #A32723) validated by manufacturer: https://www.thermofisher.com/antibody/product/Goat-anti-Mouse-IgG-H-L-Highly-Cross-Adsorbed-Secondary-Antibody-Polyclonal/A32723
* AF488 goat anti-rabbit (ThermoFisher #A32731) validated by manufacturer:

https://www.thermofisher.com/antibody/product/Goat-anti-Rabbit-IgG-H-L-Highly-Cross-Adsorbed-Secondary-Antibody-Polyclonal/A32731
* AF555 goat anti-rabbit (ThermoFisher A32732) validated by manufacturer:
https://www.thermofisher.com/antibody/product/Goat-anti-Rabbit-IgG-H-L-Highly-Cross-Adsorbed-Secondary-Antibody-Polyclonal/A32732
* AF647 goat anti-rabbit (ThermoFisher #A32733) validated by manufacturer:
https://www.thermofisher.com/antibody/product/Goat-anti-Rabbit-IgG-H-L-Highly-Cross-Adsorbed-Secondary-Antibody-Polyclonal/A32733
* AF488 goat anti-guinea pig (ThermoFisher #A21450) validated by manufacturer:
https://www.thermofisher.com/antibody/product/Goat-anti-Guinea-Pig-IgG-H-L-Highly-Cross-Adsorbed-Secondary-Antibody-Polyclonal/A-21450

WESTERN BLOTTING -- PRIMARIES
* Rabbit anti-human phospho-S6 (Cell Signaling #4857) validated by manufacturer:
https://www.cellsignal.com/products/primary-antibodies/phospho-s6-ribosomal-protein-ser235-236-91b2-rabbit-mab/4857
* Rabbit anti-histone H3 (Abcam #1791) validated by manufacturer:
https://www.abcam.com/en-dk/products/primary-antibodies/histone-h3-antibody-nuclear-marker-and-chip-grade-ab1791
* Rabbit anti-human RORalpha (clone E6G51, Cell Signaling #34639S) validated by manufacturer: https://www.cellsignal.com/products/primary-antibodies/rora-e6g5i-rabbit-mab/34639
* Mouse anti-human S6 for Westerns (Cell Signaling #2317) validated by manufacturer: https://www.cellsignal.com/products/primary-antibodies/s6-ribosomal-protein-54d2-mouse-mab/2317
* Rabbit anti-Drosophila S6 kinase, phosphoT398 (Cell Signaling #9209S) validated by manufacturer: https://www.cellsignal.com/products/primary-antibodies/phospho-drosophila-p70-s6-kinase-thr398-antibody/9209
* Mouse anti-alpha-Tubulin (University of Iowa Developmental Studies Hybridoma Bank, #AA4.3) validated by manufacturer: https://dshb.biology.uiowa.edu/AA4-3
* Rabbit monoclonal anti-GAPDH (Cell Signaling #2118L), validated by manufacturer: https://www.cellsignal.com/products/primary-antibodies/gapdh-14c10-rabbit-monoclonal-antibody/2118

WESTERN BLOTTING -- SECONDARIES
* Goat anti-mouse IgG (H+L), peroxidase conjugate (Jackson ImmunoResearch #115-035-003) validated by manufacturer:
https://www.jacksonimmuno.com/catalog/products/115-035-003
* Goat anti-rabbit IgG (H+L), peroxidase conjugate (Jackson ImmunoResearch #111-035-003) validated by manufacturer:
https://www.jacksonimmuno.com/catalog/products/111-035-003
* Goat anti-rabbit IgG (H+L), peroxidase conjugate (ThermoFisher #31466) validated by manufacturer:
https://www.thermofisher.com/antibody/product/Goat-anti-Rabbit-IgG-H-L-Secondary-Antibody-Polyclonal/31466
* Goat anti-mouse, IRDye 680RD conjugate (Li-COR #925-68070) validated by manufacturer:
https://www.licor.com/bio/reagents/irdye-680rd-goat-anti-mouse-igg-secondary-antibody
* Goat anti-rabbit, IRDye 800CW conjugate (Li-COR #925-32210) validated by manufacturer:
https://www.licor.com/bio/reagents/irdye-800cw-goat-anti-mouse-igg-secondary-antibody

Eukaryotic cell lines

Policy information about cell lines and Sex and Gender in Research

Cell line source(s)

Karpas-707H cells [Karpas, A., Dremucheva, A., and Czepulkowski, B.H. (2001). Proc Natl Acad Sci USA 98, 1799-1804] were obtained from University of Cambridge Enterprise (https://www.enterprise.cam.ac.uk/reagents/karpas-707h-human-myeloma-cell-line/). Karpas-707 cell line was established from the bone marrow of a 53 year old male with multiple myeloma: Karpas, A., et al. (1982). "HUMAN PLASMACYTOMA WITH AN UNUSUAL KARYOTYPE GROWING IN VITRO AND PRODUCING LIGHT-CHAIN IMMUNOGLOBULIN." The Lancet 319(8278): 931-933.
https://www.cellosaurus.org/CVCL_9552

Hi5 insect cells (ThermoFisher #B85502) were used in mass-spectrometric assays for in-vivo ligand identification

Authentication

No authentication tests were performed beyond expression of RORα expression using Western Blots of Karpas-707H cells

Mycoplasma contamination

Upon arrival and culturing, Karpas707H cell line tested negative for mycoplasma contamination

Commonly misidentified lines
(See ICLAC register)

Name any commonly misidentified cell lines used in the study and provide a rationale for their use.

Animals and other research organisms

Policy information about studies involving animals; ARRIVE guidelines recommended for reporting animal research, and Sex and Gender in Research

Laboratory animals

Superscripts are indicated by square brackets. Stocks obtained from the University of Indiana, Bloomington, Drosophila Stock Center (BDSC) include:
* Cg-GAL4 (Asha H et al., 2003. Genetics 163, 203-21561), #7011
* Heat-shock-GAL4(DBD)::Hr3(LBD);UAS-GFP BDSC (Palanker L et al., 2006. Development 133, 3549-3562), #28868
* phm-GAL4, TubGAL80TS -- derived from #7018
* phm-GAL4; Tub-GAL80TS -- derived from #7108 and phm[22]-GAL4, a gift from M. O'Connor, University of Minnesota)
* UAS-S6K[STED], #6913
* UAS-Tor-RNAi, #34639
* UAS-Hr3-RNAi[TRiP], #27253

* UAS-Iml1-RNAi[TRiP], #57492
* UAS-anchor-RNAi[TRiP], #51463
* UAS-RagA-B-RNAi, #34590
* UAS-RagC-D-RNAi, #32342
* pumpless (ppl)-GAL4 (Zinke I et al., Development 126, 5275-5284), #58768
* Tub-GAL80[TS]; da-GAL4, derived from #7108 and #55850
* Tub-GAL80[TS]; Tub-GAL4 #86328
* UAS-EcR-RNAi (Ni et al., 2009. Genetics 182, 1089-1100), #37058 and #37059

Fly stocks obtained from the Vienna Drosophila Resource Center include:
* UAS-anchor-RNAi, #105969
* UAS-dib-RNAi, #101117
* UAS-Hr3-RNAi[GD] (Dietzl G et al., 2007. Nature 448, 151-156), #10687
* UAS-Hr3-RNAi[GD], #12044
* UAS-Npc1a-RNAi[KK], #105405
* UAS-phm-RNAi, #108359
* UAS-S6K-RNAi, #104369
* w[1118], # 60000

unk-FireflyLuciferase (B76) (Tiebe M et al., 2015. Dev Cell 33, 272-284) was a gift from A. Teleman (DKFZ).

UAS-HR3(Full-length) and UAS-HR3(K243X) were generated in this study.

Wild animals: No wild animals were used in this study.

Reporting on sex: We only used larval animals, which exhibit minimal sexual dimorphism, so we did not concern ourselves with their sex

Field-collected samples: No field-collected animals were used in this study.

Ethics oversight: No ethics approval or oversight is required for studies in Drosophila or cultured insect or Karpas cells.

Note that full information on the approval of the study protocol must also be provided in the manuscript.

Plants

Seed stocks: not applicable

Novel plant genotypes: not applicable

Authentication: not applicable
